# A seasonal bird occurrence dataset from Karataş Lake (Burdur, Turkey) with associated NDVI and climate data, 2021 to 2022

**DOI:** 10.3897/BDJ.14.e200154

**Published:** 2026-06-17

**Authors:** Yasemin Özdemir

**Affiliations:** 1 Gölhisar Vocational School, Department of Forestry, Burdur Mehmet Akif Ersoy University, Burdur, Türkiye Gölhisar Vocational School, Department of Forestry, Burdur Mehmet Akif Ersoy University Burdur Türkiye

**Keywords:** bird diversity, drought, Jackknife, Karataş Lake, NDVI, wetland

## Abstract

**Background:**

Wetlands provide essential ecosystem services, including carbon sequestration, water purification, flood mitigation and biodiversity support. Lake Karataş has been recognised as a Wildlife Development Area since 2006. However, it is experiencing reductions in water volume and habitat size due to insufficient rainfall, water source drying and intensive agricultural practices. This study assessed ecological changes and their effects on avian diversity using remote sensing, climate data and ornithological surveys.

**New information:**

This dataset presents bird occurrence records from Lake Karataş (Burdur, Türkiye), derived from field surveys conducted in 2021–2022 using the point count method. The Normalised Difference Vegetation Index (NDVI) was derived from Sentinel-2 imagery (2017–2024) and climate data were sourced from TerraClimate. Species richness was estimated using the Jackknife method. Lake Karataş, a shallow system converted to a reservoir in 1982, is fed by the Sarıdere. In this study, 46 bird species were recorded, including the Northern Lapwing (*Vanellus vanellus*, near threatened (NT)) and Common Pochard (*Aythya ferina*, vulnerable (VU)). Remote-sensing observations from 2017 to 2024 indicated agricultural expansion, wetland contraction and marked interannual variation in lake inundation. The summer season demonstrated the highest species richness (37 species) and abundance (3785 individuals) with an NDVI of 0.1418 and an average of 32.55°C, whereas autumn's peak NDVI (0.1482) resulted in only nine species. The findings suggest that integrating NDVI, climate metrics and bird diversity indicators is crucial for restoration planning, emphasising sustainable agriculture and water management to restore ecosystem services.

## Introduction

Wetlands are essential ecosystems that offer numerous crucial services globally, including carbon storage, water purification, flood mitigation, biodiversity preservation and climate regulation (Keddy 2023, Kaya and Taşkın 2013, Yıldız 2015). These regions are of international significance, particularly as feeding, breeding and resting sites for migratory birds. Owing to its strategic location, Türkiye is home to many significant wetlands that contribute greatly to rural development from both environmental and economic standpoints (Demirel and Atalay 2017). However, these wetlands are increasingly threatened by agricultural demands, excessive water use and climate change-driven droughts (Eken et al. 2006).

Wetland preservation is crucial for maintaining biological diversity and ensuring that regional water management and agricultural growth are balanced (Ruaro and Laurance 2021, Yiğit and Keten 2024). In the specific instance of Karataş Lake, the reduction in wetland area and expansion of agricultural land are causing habitat fragmentation and a decrease in the biological diversity. This development adversely impacts the habitats of particularly vulnerable and endangered bird species, posing a threat to the Lake's ecosystem services (Kır 2005, Tokgöz and Güngör 2023).

Remote sensing technologies, particularly the Normalised Difference Vegetation Index (NDVI), are powerful tools for monitoring the vegetation health and habitat quality of wetlands (Çolak et al. 2022).

NDVI data contribute to understanding the ecological dynamics of wetlands by revealing seasonal and annual changes in the productivity of plants. The integration of NDVI analyses and climate data at Karataş Lake provides an important scientific foundation for assessing the status of wetlands in the face of agricultural pressure and climate change.

This study aimed to examine the effects of agricultural pressure, drought and environmental variability on the ecosystem of the Karataş Lake wetland area using NDVI and climate data, thereby revealing the implications of these changes for bird diversity in the area. Consequently, this will contribute to the development of sustainable strategies for wetland management and conservation in the future.

## Project description

### Study area description

**Figure 1.**
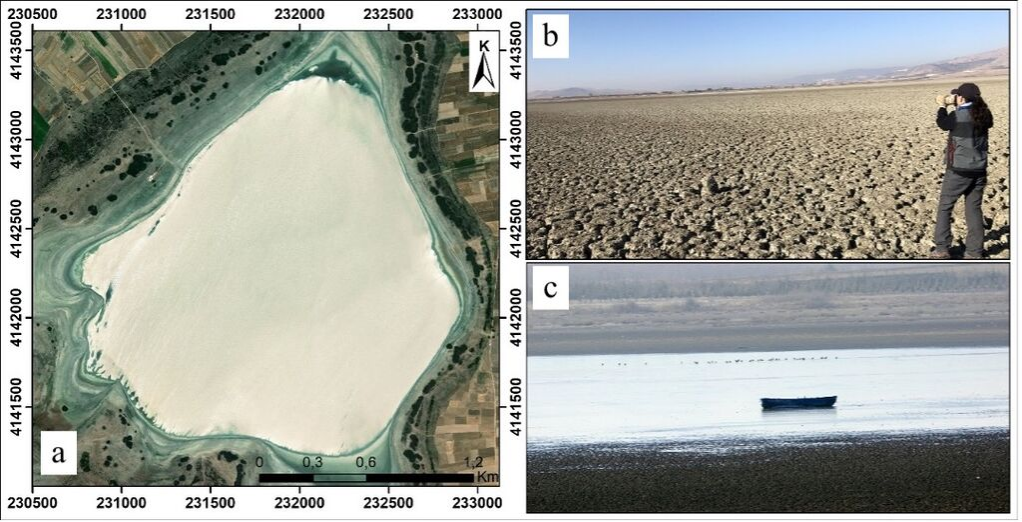
Overview of Karataş Lake (Burdur, Türkiye) based on remote sensing and field observations. **a** Satellite image of the study area showing lake boundaries and surrounding habitats; **b** Field photograph of the exposed lake-bed under dry conditions; **c** Field photograph of the inundated area with observed bird assemblages.

Karataş Lake, located in the Karamanlı District of Burdur Province (Türkiye), is one of the important wetlands in the Lakes Region and has been a feeding and breeding ground for migratory birds for several years (Fig. 1). Geographically located at coordinates (37°23’N, 29°58’E), the Lake plays a critical role in the ecological balance of the region, covering a surface area of 2.2 km², measuring 2134 m in width and 2087 m in length and having an average depth of 2 m (Tuncer 2021). However, in recent years, due to low rainfall, drying up of water sources and intensification of agricultural activities, the lake's water volume has decreased and its ecosystem functions have been significantly impaired (Eken et al. 2006, General Directorate of Water Management 2021).

**Figure 2.**
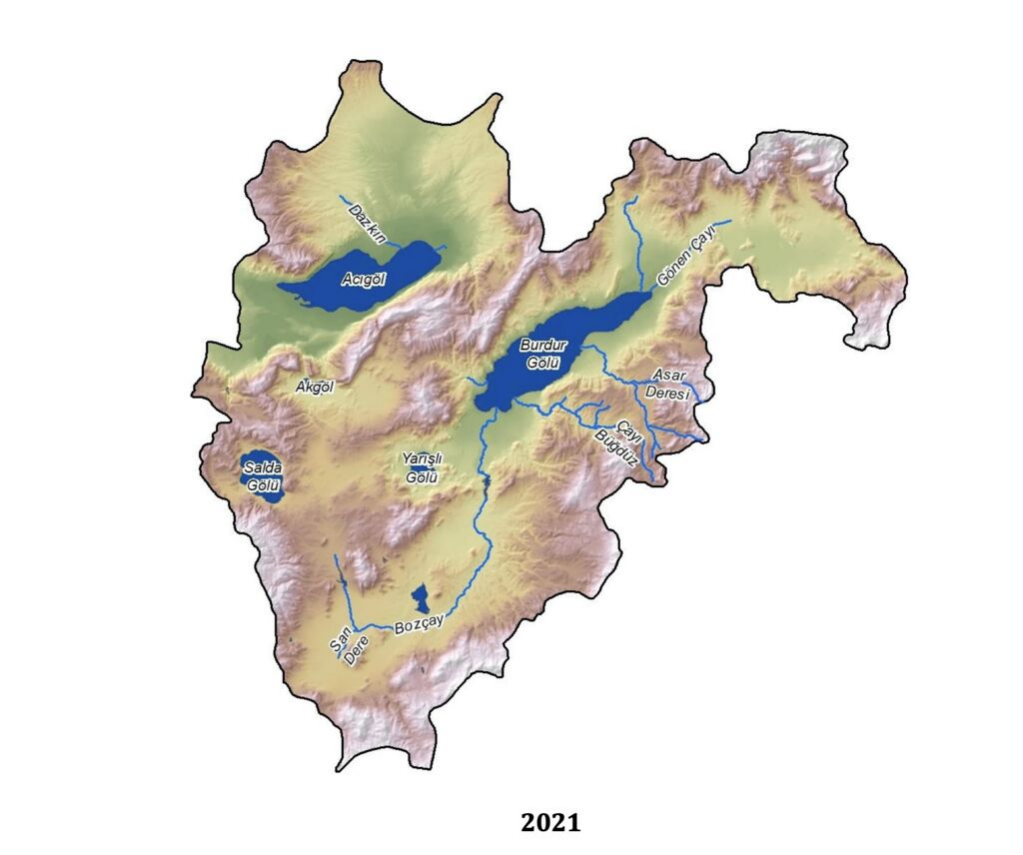
Burdur Basin River Basin Management Plan Strategic Environmental Assessment Final Report 2021 (General Directorate of Water Management 2021).

The most important river feeding Lake Karataş is Sarıdere, which flows through water channels that pass through Hüyükköy (Fig. 2). Lake Karataş, which is on the list of international wetlands, was declared a Wildlife Development Area in 2006 (General Directorate of Water Management 2021).

### Design description

The field data for the study were collected during the 2021-2022 period, whereas the satellite images and climate data covered the years 2017-2024. This time frame is critical for monitoring long-term changes in wetlands and distinguishing the effects of agricultural pressure and climate variability.

## Sampling methods

### Study extent

The study was conducted at Karataş Lake and its surrounding wetland habitats located in Burdur Province, south-western Türkiye. The area is part of the Lakes Region and represents an important freshwater ecosystem supporting diverse bird species. The study covered seasonal observations within the wetland.

### Sampling description

Bird observations were carried out through direct field surveys during different seasons. Standard ornithological observation techniques were used, including visual identification using binoculars and field guides. Two commonly used methods in avian surveys are line transects and point counts (Dobinson 1976, Bibby et al. 1992a, Bibby et al. 1992b). While the line transect method is generally used to assess bird movement and distribution across large areas, the present study employed the point count method to evaluate species diversity and individual abundance at fixed locations. For each observation, species identification, number of individuals, observation date and geographic coordinates were recorded. The dataset represents presence-only (occurrence) data and does not include absence records. The sampling approach aimed to capture seasonal variation in bird diversity and distribution.

Bird surveys were conducted using the point count method at a single observation station established within Karataş Lake. Observations were carried out once in each season (spring, summer, autumn and winter), resulting in a total of four sampling events. Each survey lasted 10 minutes, corresponding to the sampleSizeValue reported in the dataset metadata. During each observation period, all birds detected visually or acoustically were recorded and identified to species level.

### Quality control

Data quality was ensured through careful species identification using standard field guides and observation techniques. Geographic coordinates were recorded using GPS devices and verified for accuracy. Taxonomic names were standardised according to accepted scientific nomenclature.

### Step description

Field data were collected during seasonal surveys and recorded in tabular format. Geographic coordinates were converted into decimal degrees (WGS84). The dataset was cleaned by removing incomplete and duplicate records. Taxonomic names and metadata were standardised according to Darwin Core terms. The final dataset was published through the GBIF Integrated Publishing Toolkit (IPT).

In this study, remote sensing and field observation techniques were used to examine the ecosystem dynamics of Lake Karataş and its surroundings. The health and seasonal changes in the vegetation cover of wetlands were assessed using the Normalised Difference Vegetation Index (NDVI). NDVI data (Suppl. material 2) were derived from multispectral images obtained from the Sentinel-2 satellite, which provided reliable information on vegetation density and health (Huete et al. 2002, Pettorelli et al. 2005). NDVI analyses were used to reveal seasonal and annual changes in wetland vegetation productivity.

Temperature and precipitation data (Suppl. material 1), as climatic variables, were obtained from the TerraClimate database (Abatzoglou et al. 2018). These data were evaluated as seasonal averages to analyse the effects of regional climate variability on wetland ecosystems. The effects of climate data on NDVI and bird diversity were comparatively examined. Bird diversity was calculated using Jackknife analysis in the PAST 4 software.

In the data analysis (Correlation analysis), the relationships between seasonal changes in NDVI, climate data, bird species richness and individual numbers were evaluated statistically. Multivariate analysis techniques, particularly Principal Component Analysis (PCA), have been used to reveal the effects of environmental variables on bird populations (Jolliffe 2014). This allowed for a more comprehensive interpretation of the dynamics between habitat quality and environmental pressures.

## Geographic coverage

### Description

Karataş Lake is located in Burdur Province, south-western Türkiye. The dataset covers the Lake and its surrounding wetland habitats used by resident and migratory bird species.

## Taxonomic coverage

### Description

In this study, we covered the following taxonomic groups: Orders Columbiformes, Charadriiformes, Podicipediformes, Anseriformes, Phoenicopteriformes, Passeriformes, Strigiformes, Pelecaniformes, Coraciiformes, Falconiformes, Accipitriformes and families: Fringillidae, Scolopacidae, Charadriidae, Recurvirostridae, Laridae, Sylviidae, Corvidae, Accipitridae, Columbidae, Hirundinidae, Strigidae, Motacillidae, Muscicapidae, Alaudidae, Phoenicopteridae, Podicipedidae, Falconidae, Ardeidae, Anatidae, Meropidae and Pelecanidae.

## Temporal coverage

**Data range:** 2021-7-15 – 2022-4-27.

## Usage licence

### Usage licence

Open Data Commons Attribution License

## Data resources

### Data package title

Seasonal bird sampling event dataset from Karataş Lake (Türkiye)

### Resource link

https://doi.org/10.15468/s4k6t2

### Number of data sets

1

### Data set 1.

#### Data set name

Seasonal bird sampling event dataset from Karataş Lake (Türkiye)

#### Data format

Darwin Core Archive

#### Download URL

https://www.gbif.org/dataset/adba9430-6ea2-492d-8d35-1ef26f76fe18

#### Description

The dataset consists of bird occurrence records collected from Karataş Lake and its surrounding habitats (Özdemir 2026). Each record represents an individual observation and includes information such as scientific name, geographic coordinates (decimal latitude and longitude), observation date and individual count. The data are structured according to Darwin Core standards and organised in a tabular format suitable for biodiversity data sharing platforms such as GBIF. The dataset can be used for ecological analysis, species distribution studies and assessments of the relationship between bird diversity and environmental variables such as NDVI. Users can integrate this dataset with other environmental or spatial datasets for advanced ecological modelling and conservation research.

The data in this occurrence resource has been published as a Darwin Core Archive (DwC-A), which is a standardised format for sharing biodiversity data as a set of one or more data tables.

This IPT archives the data and thus serves as the data repository. The data and resource metadata are available for download in the downloads section. The versions table lists other versions of the resource that have been made publicly available and allows tracking changes made to the resource over time.

**Data set 1.** 

Column label	Column description
id (Event Core, Occurrence Extension)	Unique identifier.
eventID (Event Core, Occurrence Extension)	A unique identifier linking events and occurrences.
samplingProtocol (Event Core)	The sampling method used.
samplingEffort (Event Core)	Seasonal point count survey.
occurrenceID (Occurrence Extension)	A unique identifier for the occurrence record within the dataset.
eventDate (Event Core)	Date the record was collected.
locationID (Event Core)	An identifier of the location.
country (Event Core)	Country in which the survey was conducted.
countryCode (Event Core)	ISO code of the country.
decimalLatitude (Event Core)	The geographic latitude, in decimal degrees.
decimalLongitude (Event Core)	The geographic longitude, in decimal degrees.
geodeticDatum (Event Core)	The reference point for the various coordinate systems used in mapping the earth.
coordinateUncertaintyInMetres (Event Core)	Uncertainty of the coordinates, in metres.
sampleSizeValue (Event Core)	Duration of the bird observation.
sampleSizeUnit (Event Core)	Unit of measurement for the sampling effort reported in sampleSizeValue.
eventTime (Event Core )	Time when the sampling event took place.
basisOfRecord (Occurrence Extension)	The specific nature of the data record.
occurrenceStatus (Occurrence Extension)	A statement about the presence or absence of a taxon at a location.
phylum (Occurrence Extension)	Phylum name in which the taxon is classified.
class (Occurrence Extension)	Class name in which the taxon is classified.
order (Occurrence Extension)	Order name in which the taxon is classified.
family (Occurrence Extension)	Family name in which the taxon is classified.
scientificName (Occurrence Extension)	The full scientific name.
taxonRank (Occurrence Extension)	The taxonomic rank of the most specific name in the scientificName.
startDayOfYear (Event Core)	Sequential day number within the year.
eventRemarks (Event Core)	Additional comments or notes about the sampling event.
locality (Event Core)	Specific description of the sampling locality.
type (Event Core)	Nature or type of the resource; commonly Dataset.
ownerInstitutionCode (Event Core)	Code or name of the institution that owns the dataset.
individualCount (Occurrence Extension)	Number of individuals observed.
organismQuantityType (Occurrence Extension)	Type of quantity recorded.
kingdom (Occurrence Extension)	Taxonomic kingdom of the organism; for birds.
recordedBy (Occurrence Extension)	Name of the person(s) who recorded the occurrence.

## Additional information

### Results and Discussion

**Figure 3.**
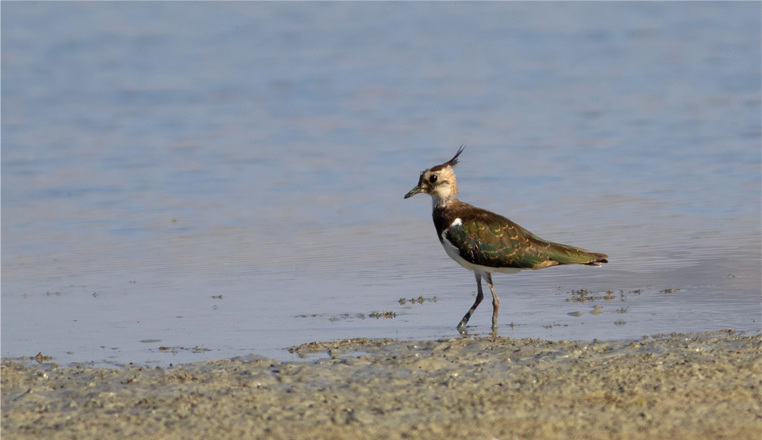
Photograph of *Vanellus vanellus* taken in a study area classified as Near Threatened (NT). Photo: Yasemin ÖZDEMİR.

As one of the most important wetlands in terms of ornithofauna, the Lake is home to numerous native and migratory bird species. During the inventory studies, 46 species belonging to 11 orders and 21 families were identified in the study area. Amongst the identified species, the Northern Lapwing (*Vanellus vanellus*) is classified as Near Threatened (NT) (Fig. 3) and the Common Pochard (*Aythya ferina*) as Vulnerable (VU) according to the IUCN Red List.

**Figure 4.**
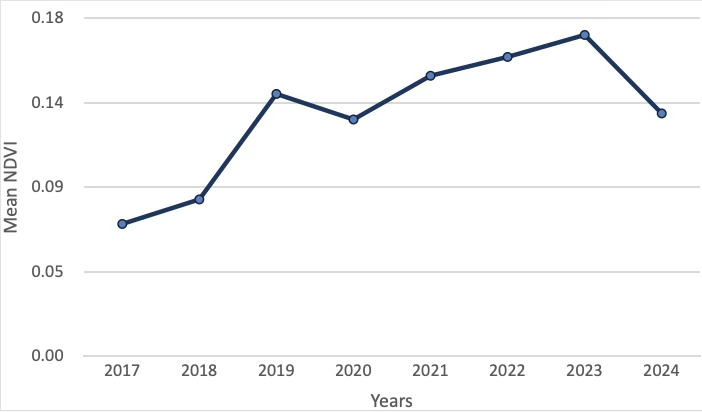
Temporal variation of average NDVI values in Karataş Lake during the summer period of 2017–2024.

Analyses show that agricultural areas have increased over the years (2017-2024), whereas wetland surface areas have shrunk significantly (Fig. 4). Although the Lake area that dried up in 2021 began to retain water again in 2022, it had not yet regained its former ecological function by the time of our last survey, mainly due to agriculture-related pollution and water scarcity. The protection and sustainable management of wetlands are critical for preventing such losses (Quintana 2018). The continuity of ecosystem services will be possible through sound management of these areas. Wetland protection is vital for maintaining biodiversity and human life (Çelik and Gülersoy 2013). Therefore, effective protective measures should be implemented at both local and national levels.

**Figure 5.**
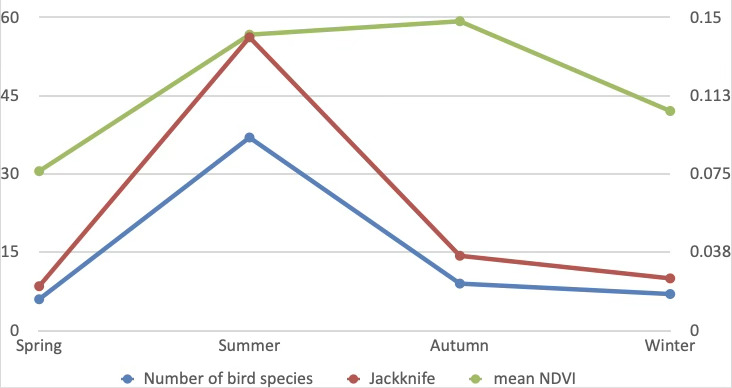
Number of bird species, NDVI and Jackknife values by season (2021–2022).

During summer, the highest bird diversity (56.2) was recorded, with 37 bird species and 3785 individual birds (Fig. 5, Table 1). In this season, NDVI (average 0.1418) and temperature (average 32.55°C) are at their highest levels, whereas precipitation is at its lowest. In autumn, although NDVI reached its highest level at an average of 0.1482, the number of bird species was only nine. It was determined that NDVI data alone were not decisive and that other environmental factors also affected habitat suitability (Figs 5, 7).

**Figure 6.**
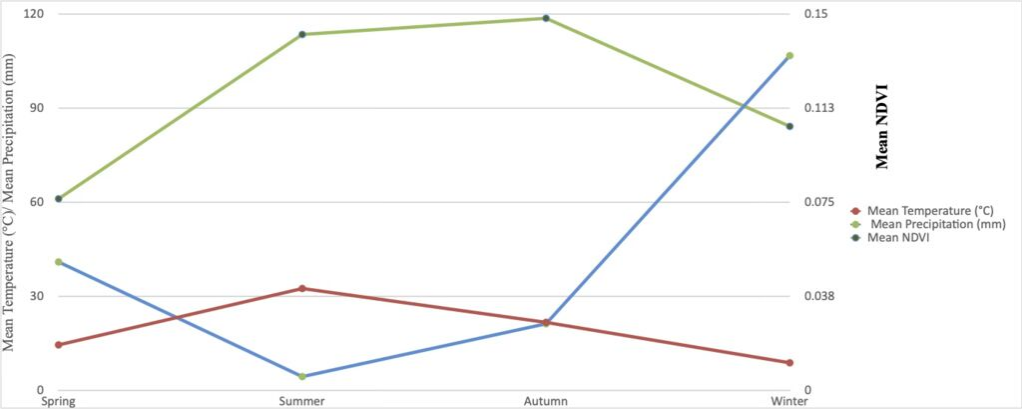
Seasonal averages between July 2021 and April 2022.

Exploratory comparison of the seasonal data suggested that bird species richness, abundance and Jackknife richness estimates tended to vary in parallel with temperature and, to a lesser extent, NDVI. Rainfall showed an opposite seasonal pattern in relation to these biodiversity metrics. As the analysis was based on only four seasonal observations, no formal significance testing was applied and the observed patterns should be interpreted as descriptive indications rather than statistically confirmed relationships. The Normalised Difference Vegetation Index (NDVI) is an indicator that reflects the density and health of vegetation and, in this context, it provides information about the habitat quality of wetlands (Fig. 6).

**Figure 7.**
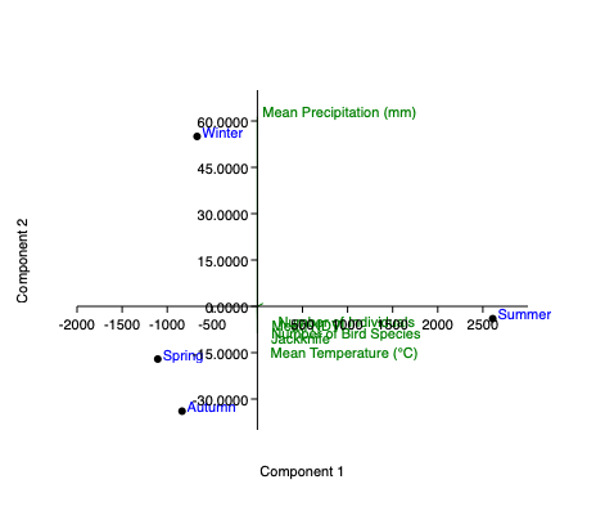
Bird Observation Data and Environmental Variables by Season (Principal Component Analysis (PCA)).

The summer season had the highest number of birds. The winter season is significant in terms of the highest rainfall and the effects of environmental factors on species diversity (Fig. 7). Autumn was particularly distinguished by differences in Jackknife and temperature-related variables.

Karataş Lake, a natural lake converted into a reservoir in 1982 (Çetin 2009), has a volume of 65.3 hm³. The lake's surface area has significantly decreased, particularly because of irrigation (Çolak et al. 2022).

The water loss and habitat shrinkage observed in our study are associated with several problems. Wetlands are critically important for the sustainability of migration routes, providing feeding, shelter and resting opportunities during bird migration. The successful completion of bird migrations largely depends on the existence of these areas and their protection from human activity. The degradation or loss of wetlands along migration routes negatively impacts the energy balance of bird populations, reducing migration success and threatening the long-term survival of the species.

Karataş Lake (Burdur) is an important freshwater reservoir in the Lakes Region. Despite its small surface area, it has high ecological and ornithological value and contributes positively to the surrounding agricultural and fishing activities (Çetin 2009). It is emphasised that the Lake must be carefully protected and urgent measures must be taken to ensure the sustainability of wildlife in the region (Kır 2005, Çetin 2009).

This study has several limitations that should be considered when interpreting the results. Bird surveys were conducted during 2021–2022 and, therefore, represent a relatively short temporal sampling window. In contrast, NDVI analyses covered a longer period (2017–2024), providing broader information on vegetation dynamics in the study area. Furthermore, the dataset consists of occurrence records only and does not include absence data, which limits the ability to infer species distributions or habitat suitability. Consequently, the findings should be interpreted as evidence of seasonal patterns and associations rather than definitive causal relationships.

### Conclusions

The ecological balance between Karataş Lake and the surrounding agricultural landscape and wetland habitats creates a critical habitat for bird populations. NDVI analyses have revealed changes in vegetation health, particularly during periods of increased agricultural intensity. These changes directly affect the seasonal distribution of bird species in the Lake ecosystem. Habitat fragmentation around the Lake, which is under pressure from agricultural activities, threatens both biodiversity and water quality. Therefore, the sustainable use of Lake Karataş is possible through continuous monitoring using remote-sensing techniques and integrated land management practices (Çolak et al. 2022, Tokgöz and Güngör 2023, Güneş Tigen and Özcan 2025).

Lake Karataş is an ecosystem that plays a key role in regional wetland conservation and supports a rich aquatic and terrestrial biodiversity. Analyses using NDVI and other satellite-based indices have revealed that the vegetation cover density around the Lake has varied over the years and that these changes are associated with human-induced land use (Özvan et al. 2023, Güneş Tigen and Özcan 2025).

In particular, the gradual reduction of reed beds and natural habitats threatens the populations of frogs, reptiles and fish species living in the Lake (Kır 2005). These findings highlight the need for urgent protection and management measures to maintain the ecological integrity of Karataş Lake.

The use of remote sensing techniques for monitoring and managing wetlands is of great importance for conserving ecosystem services (Özvan et al. 2023). These methods can contribute to the development of sustainable conservation strategies by providing the ability to effectively assess temporal changes in wetland condition. In this context, in addition to remote sensing methods, the participation and awareness of local communities play an important role in wetland conservation.

To restore the ecological function of Lake Karataş, sustainable agricultural practices, planned water resource use and wetland management should be promoted. The integrated use of indicators, such as NDVI and bird diversity, provides a scientific basis for future wetland restoration efforts. This study highlights the importance of monitoring NDVI and climatic data in conjunction with bird diversity for effective wetland management.

## Supplementary Material

A9BD5D33-7868-512A-983A-7B57DF852800 10.3897/BDJ.14.e200154.suppl1 Supplementary material 1 NDVI and climatic data for Lake Karataş. Data type environmental Brief description This dataset includes average seasonal NDVI values and associated climatic variables (average temperature and precipitation) for Lake Karataş. File: oo_1605961.xlsx https://binary.pensoft.net/file/1605961 Yasemin Özdemir

4CBC83B7-02DE-512B-AE2C-FD400081B154 10.3897/BDJ.14.e200154.suppl2 Supplementary material 2 Long-term (2017–2024) NDVI data for Lake Karataş. Data type environmental Brief description This dataset includes long-term (2017–2024) average NDVI values for Lake Karataş, derived from satellite imagery to assess temporal variation in vegetation dynamics. File: oo_1605979.xlsx https://binary.pensoft.net/file/1605979 Yasemin Özdemir

## Figures and Tables

**Table 1.** Number of bird species, NDVI and Jackknife values by season (2021–2022).

Season	Number of bird species	Jackknife	mean NDVI
Spring	6	8.5	0.076377
Summer	37	56.2	0.141768
Autumn	9	14.33	0.148206
Winter	7	10	0.105202
